# Gene expression profiles of YAP1, TAZ, CRB3, and VDR in familial and sporadic multiple sclerosis among an Iranian population

**DOI:** 10.1038/s41598-021-87131-z

**Published:** 2021-04-08

**Authors:** Sheyda Khalilian, Zohreh Hojati, Fariba Dehghanian, Vahid Shaygannejad, Seyedeh Zahra Hosseini Imani, Majid Kheirollahi, Mehdi Khorrami, Omid Mirmosayyeb

**Affiliations:** 1 Division of Genetics, Department of Cell and Molecular Biology and Microbiology, Faculty of Biological Sciences and Technologies, University of Isfahan, 81746-73441 Isfahan, Iran; 2 Research Institute for Primordial Prevention of Non-Communicable Disease and Department of Genetics and Molecular Biology, School of Medicine, Isfahan University of Medical Sciences, Isfahan, Iran; 3 Isfahan Neuroscience Research Center, Isfahan University of Medical Sciences, Isfahan, Iran

**Keywords:** Genetics, Molecular biology, Neuroscience

## Abstract

Alterations in the regulatory mechanisms that control the process of myelination in the nervous system, may lead to the impaired myelination in the Multiple sclerosis. The Hippo pathway is an important mediator of myelination in the nervous system and might contribute to the pathophysiology of MS. This study examined via qPCR the RNA expression of *YAP1*, *TAZ*, and *CRB3* as the key effectors of the Hippo pathway and also, *VDR* in the peripheral blood of 35 sporadic, 37 familial MS patients; and also 34 healthy first-degree relatives of the familial MS patients (HFR) and 40 healthy individuals without a family history of the disease (control). The results showed the increased expression of *VDR* in the sporadic group, as compared to other groups. There was also an increased expression of *TAZ* in the familial and HFR groups, as compared to the control group. The familial and sporadic patients displayed a significantly lower level of expression of *YAP1* in comparison to the HFR group. The increased expression level in the sporadic patients and control group, as compared to the HFR group, was seen in *CRB3*. We also assessed different clinical parameters and MRI characteristics of the patients. Overall, these findings suggest that Hippo pathway effectors and also *VDR* gene may play a potential role in the pathophysiology of the sporadic and familial forms of MS. Confirmation of different gene expression patterns in sporadic and familial MS groups may have obvious implications for the personalization of therapies in the disease.

## Introduction

Multiple sclerosis (MS) is a chronic inflammatory demyelinating disease of the central nervous system, with variable axonal and neuronal injury, leading to the formation of multiple focal areas of myelin loss within the CNS, called plaques^1,2^. The function of the nervous system relies on the ability of the nerve fibers to transmit signals between different areas of the brain, and between the brain and the rest of the nervous system. The speed of the signal conduction in these fibers is regulated by myelin, a cholesterol-rich extension of oligodendrocytes and the Schwann cells plasma membrane^3,4^. Although most MS cases occur sporadically; a significant number of patients are related by family^5^. Onset age and imaging characteristics are found to be the two different features among sporadic and familial MS^6^. Based on twin studies, the concordance rate in monozygotic twins is 24%, as compared to 3% for the dizygotic twins^7^. This is further supported by the fact that the familial MS, families with two or more MS cases, accounts for 12.5% of all MS cases^8^. The lifetime risk of MS for the general population is about 0.2% ^9^. First-degree relatives have a risk of approximately 3–5%, and 15–25 times higher relative risk, as compared to the background population^8^. Given diseases pattern among relatives, physicians could determine whether an individual or future generations may be at an increased risk of developing a particular disease^10^. Despite many years of intensive research, key aspects of multiple sclerosis etiology and pathogenesis still remain unanswered^11,12^. It is considered that complex gene-environment interactions lead to the manifestation of the disease^13^. Certain genetic factors are thought to confer risk to susceptibility for a specific disease that may share pathophysiological mechanisms. Because of this, an association between MS and other autoimmune diseases (AIDs) has also been suggested^14^.

The identification of genes induced during the myelination process and activated during oligodendrocytes differentiation is still far from complete^15^. There is extensive evidence suggesting that nerves lacking *YAP/TAZ* are unable to myelinate and experience a global dysregulation of transcription^16–18^. *YAP/TAZ* are two transcriptional activators of the Hippo signaling pathway and play important roles in controlling organ size, cell death, and cell proliferation and differentiation^19,20^. *Crb3*, as an inhibitor of *YAP/TAZ* through the Hippo pathway, is active at myelinating glial cell microvillus and stimulates the Hippo pathway through FRMD6, which is also located in microvillus^21,22^. In the myelinating glial cells, *YAP/TAZ* silencing reduces both myelin sheath length and thickness and its presence is necessary to mediate the *Crb3* effect on the myelin sheath length^23^. Taking together, these data indicate that nerve stretching during body growth increases myelin sheath length through the activation of *YAP/TAZ* or the silencing of *Crb3*, in the myelinating glial cells, promoting myelination via transcriptional activity^24^. Increasing evidence suggests that *TAZ* regulates the expression of the vitamin D receptor (*VDR*), by regulating the p53 transcriptional activity. Indeed, the knockdown of *TAZ* could decrease the expression of *VDR*^25^. Considering that 1,25(OH)2D, the active form of vitamin D, can cross the blood–brain barrier and enter the CNS, it seems plausible to assume that *VDR* is expressed in the neurons and plays an essential role in myelination^26^. Notably, oligodendrocytes express *VDR*, and 1,25(OH)2D depletion results in the reduced differentiation into oligodendrocytes with increased apoptosis, axonal injury, and demyelination^27^. 1,25(OH)2D and *VDR,* therefore, positively regulate oligodendrocytes differentiation and myelination^28^.

Therefore, to evaluate regulators that are essential for the myelination process, we examined the expression level of *VDR*, *TAZ*, *YAP1*, and *CRB3* genes in the familial and sporadic MS patients versus healthy individuals with and without a family history of the disease. Patients were divided into two sporadic and familial groups to investigate the factors involved in the familial aggregation of the disease. Also, healthy first-degree relatives of familial patients were involved in the study to identify a distinct type of gene expression pattern that indicates a predisposition to MS. Besides, the assessment was performed to evaluate whether the expression of these genes would be related to the clinical features of MS (disease activity, neurological disability, disease duration, and expanded disability status scale). The usefulness of these molecules as biomarkers for discriminating different clinical forms of MS was also examined.

## Materials and methods

### Patients

Blood samples were obtained from patients referred to the MS clinic of Kashani Hospital, affiliated with Isfahan University of Medical Sciences between June 2018 and September 2019, and were collected in the EDTA containing vials. Totally, 72 MS patients including 35 sporadic patients and 37 patients with a family history of MS (familial cases), and 74 Healthy controls including 34 healthy first-degree relatives of the familial MS patients (HFR) and 40 healthy individuals without a family history of MS (control), made the samples. MS diagnosis was established according to the McDonald criteria^29^. IFN-Beta was used for the treatment of patients. Those with coexistence of other neurological symptoms were excluded from the study. The control group participants were matched to the patients for age and sex. In addition, they had no known neurological disorder, family history of autoimmune or chronic inflammatory diseases, and were only admitted for health check. Moreover, we have excluded healthy individuals who had a first-degree relative with a history of neurological disorder. Patients were evaluated for the expanded disability status scale (EDSS), and brain and spinal magnetic resonance imaging (MRI) findings by their treating physicians. All MRI evaluations were performed by a 1.5 T Magnetom Avanto (Siemens, Erlangen, Germany) using a head and neck coil. The MRI protocol included sagittal STIR (TR = 4000 ms, TE = 70 ms, flip angle: 180°, inversion time: 160 ms, FOV = 220, NEX = 2), sagittal T2W FSE (TR = 2500 ms, TE = 102 ms, flip angle: 90°, FOV = 240, NEX = 2), and axial T2W FSE images (FOV = 180, NEX = 1). All images were assessed by an experienced neurologist. The study was performed in accordance with the seventh edition of the Helsinki declaration and approved by the ethics committee of the University of Isfahan, and all participants gave their informed consent.

### Total RNA extraction and cDNA synthesis

Total RNA was extracted from the peripheral blood of patients and healthy controls using the FavorPrep Blood/Cultured Cell Total RNA Mini Kit (Favorgen, Taiwan) by following the manufacturer’s instructions. The total RNA was eluted with nuclease-free water, and the samples were stored at − 70 °C. The RNA concentration was determined using NanoDrop ND-100 (Thermo Scientific, USA). Total RNA was reverse transcribed to cDNA in a 10 μl reaction volume using the PrimeScript RT reagent Kit (Takara, Japan), with the standard protocol. RT reaction mixture contained 2 μl of 5 × PrimeScript Buffer, 0.5 μl of random hexamer primers, 0.5 μl of Oligo dT Primers, 0.5 μl of PrimeScript RT Enzyme Mix I, and 6.5 μl of the extracted RNA solution plus RNase-free water. The samples were incubated as follows: 15 min at 37 °C, and 5 s at 85 °C. cDNA samples were stored at − 20 °C.

### Quantitative real time-PCR

Forward and Reverse primers of the target genes were designed by the NCBI online tool and Oligo 7 Primer Analysis Software (synthesized by the Bioneer Company). The sequences of all primers are given in Supplementary Table S1. All oligonucleotide primers were designed to span the intronic sequences. Real-time PCR was performed using RealQ Plus 2 × Master Mix Green (Ampliqon, Denmark) by Bio-Rad and Applied Biosystems machines. All samples were run in duplicates. Quantitative-PCR reactions were run under standard conditions: initial denaturation at 95 °C for 15 min, after which 40 amplification cycles of 30 s denaturation at 95 °C and 30 s annealing and extension in 72 °C could be performed. PCR products were run on 2% agarose gel, producing the amplicons of the expected size (Supplementary Fig. S1).

The gene expression data were analyzed using the comparative Ct method (ΔΔCt). Calculation of the relative amounts of genes was performed using the *ACTB* reference gene with one healthy control sample used as a calibrator.

### Statistical analysis

Statistical analysis was performed using the SPSS 26 software package (SPSS Inc. 2019. Chicago) and Graph Pad Prism 8 (Graph Pad Prism Software, Inc. San Diego CA, USA). Typically, the one-way ANOVA was used to test for expression differences among four study groups. Independent sample t-test was used to compare the expression variations between MS patients and control groups. Furthermore, the Spearman correlation coefficient was used to examine the association of the relative gene expression levels versus clinical parameters. Finally, by plotting receiver operating characteristic (ROC) curves and measuring the area under the curve (AUC), the potential of each gene as a biomarker, in each defined group, was specified. The level of significance was set at 0.05.

## Results

### Clinical and MRI data

We classified all individuals who participated in this study in four different groups. The population consisted of 35 sporadic patients, 37 familial patients, 34 HFRs, and 40 controls. Patients were largely female (71.4%, 75.67%) with an average age of 38.00 ± 10.59, 36.26 ± 9.66 years in sporadic and familial groups, respectively. Also, most of the population of healthy participants were female (55.88%, 72.5%) with a mean age of 38.47 ± 9.28, 37.38 ± 15.94 years in HFR and control group, respectively. There were no significant differences regarding either age (p = 0.387) or sex (p = 0.165) between four groups.

The onset age and EDSS results were not meaningfully different between sporadic and familial groups. The proportion of parental consanguinity was low among both patient groups. All of the patients in both groups were with Persian ethnicity and most of them were diagnosed with the relapsing–remitting type of MS (RR-MS). The family history of AIDs was significantly higher in the familial group, as compared to the sporadic one (p-value: 0.016). However, the majority of the patients were not diagnosed with a family history of AIDs. The MRI findings, such as the lesion load, brain atrophy, the number of cervical plaques, and Longitudinally extensive transverse myelitis (LETM), were not significantly altered in both sporadic and familial groups. Table 1 presents the detailed characteristics of all patient and healthy groups.

**Table 1:** Demographic and clinical characteristics of the MS patients and healthy subjects.

Features	Sporadic patients N = 35	Familial patients N = 37	HFR group N = 34	Control group N = 40	p-value
**Sex**					
Male	10 (28.6%)	9 (24.32%)	15 (44.11%)	11 (27.5%)	0.165
Female	25 (71.4%)	28 (75.67%)	19 (55.88%)	29 (72.5%)	
Age	38.00 ± 10.59	36.26 ± 9.66	38.47 ± 9.28	37.38 ± 15.94	0.387
EDSS	2.11 ± 2.02	1.95 ± 2.27	–	–	0.541
Onset age	30.88 ± 10.13	28.97 ± 8.06	–	–	0.435
Time from first symptoms (years)	1.82 ± 3.92	3.30 ± 12.80	–	–	0.063
Number of attacks through the year	1 ± 1	1 ± 1	–	–	0.490
**MS type**					
RRMS	19 (55.9%)	23 (71.9%)	–	–	0.037
SPMS	5 (14.7%)	8 (25.0%)	–	–	
PPMS	2 (5.9%)	0 (0.0%)	–	–	
Other	8 (23.5%)	1 (3.1%)	–	–	
**Consanguinity**					
Yes	6 (17.1%)	7 (29.2%)	–	–	0.274
No	29 (82.9%)	17 (70.8%)	–	–	
**Brain atrophy**					
Yes	10 (30.3%)	7 (28.0%)	–	–	0.947
No	23 (69.7%)	18 (72.0%)	–	–	
**Lesion load**					
Low	9 (28.1%)	5 (20.8%)	–	–	0.419
Medium	13 (40.6%)	14 (58.3%)	–	–	
High	10 (31.3%)	5 (20.8%)	–	–	
**Cervical plaques**					
0	6 (19.4%)	4 (17.4%)	–	–	0.806
1	8 (25.8%)	8 (34.8%)	–	–	
1–3	7 (22.6%)	6 (26.1%)	–	–	
> 3	10 (32.3%)	5 (21.7%)	–	–	
**LETM**					
Yes	6 (19.4%)	7 (31.8%)	–	–	0.299
No	25 (80.6%)	15 (68.2%)	–	–	

*Values are Mean ± SD or Number (Percent).

### Assessment of the relative gene expression levels of *VDR*, *TAZ*, *YAP1*, and *CRB3* among genetically separated groups

**Figure 1:**
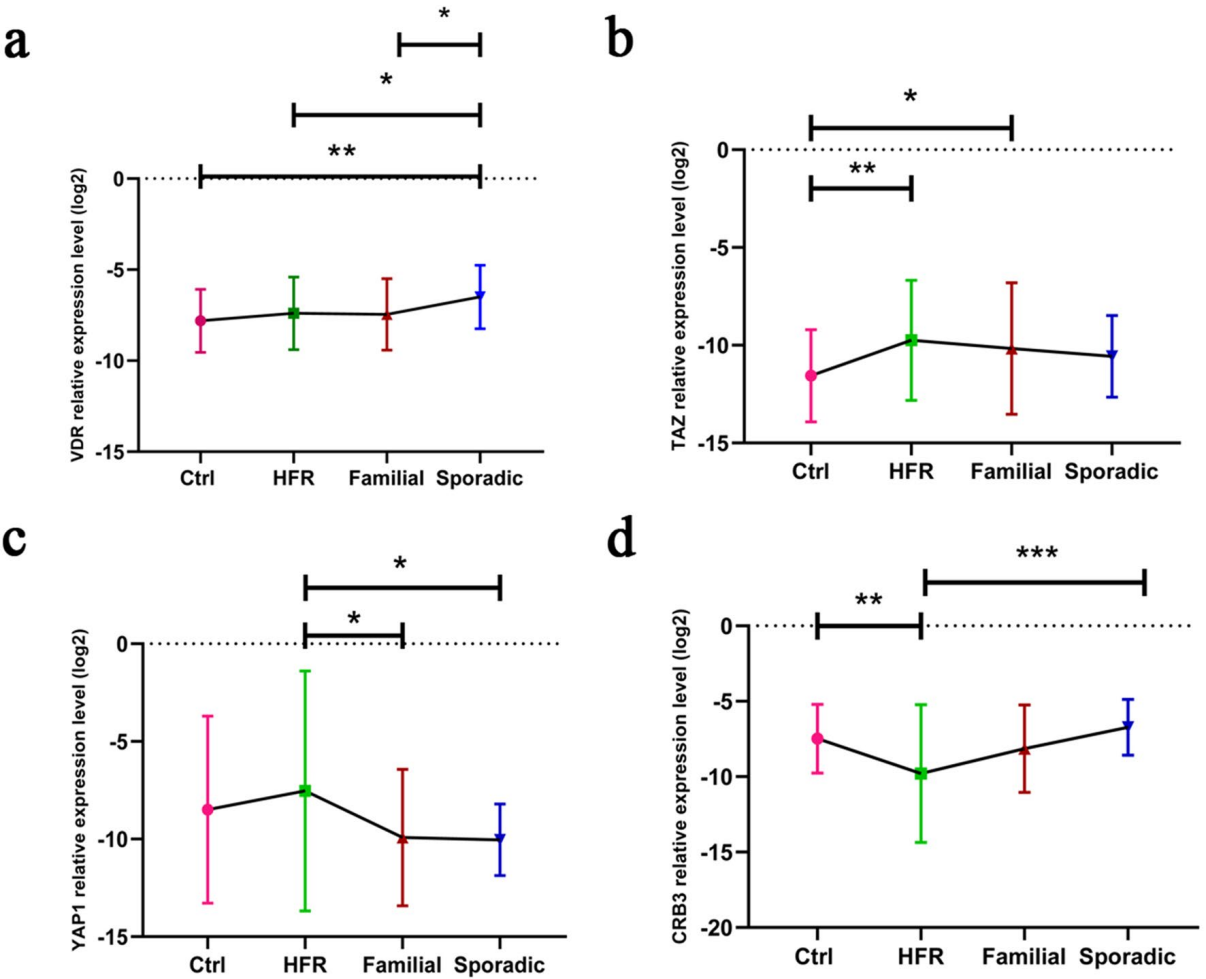
Differential expression level of (**a**) *VDR*, (**b**) *TAZ*, (**c**) *YAP1*, and (**d**) *CRB3* among genetically separated groups and control group. Ctrl = control group, HFR = healthy first-degree relatives of the familial group. Bars represent the standard error of the mean. (*p < 0.05, **p < 0.01, ***p < 0.001).

Comparison of the relative expression of *VDR* in the four study groups showed the increased expression in the sporadic group, as compared to the HFR one (p-value: 0.04), as well as the control group (p-value: 0.002). This increase in expression, as expected, was seen in the sporadic patients, as compared to the familial group (p-value: 0.02) (Fig. 1a). In the case of the *TAZ* gene, the relative expression comparison among the four groups showed an increased expression in the familial group, as compared to the control group (p-value: 0.02). There was also a significant increase in the expression level of the HFR group when compared with the control one (p-value: 0.004). As expected, this increase in expression was also seen in the sporadic patients, as compared to the control group, but it was not statistically significant (p-value: 0.12) (Fig. 1b). Comparison of the relative expression of *YAP1* in the four groups showed a decrease in the expression of familial and sporadic patients, as compared to the HFR group (p-value 0.02, 0.01 respectively). Regarding these differences in the expression level, the control group was expected to show differential relative expression in comparison with the HFR group. There was such a difference in the expression level, but it was not statistically significant (Fig. 1c). The increased expression level in sporadic patients and control group, as compared to the HFR one, was also seen in *CRB3* (p-value: 0.0003, 0.007 respectively); however, there was not a significant differential expression, as expected, in the familial group versus sporadic or HFR groups (Fig. 1d).

**Figure 2:**
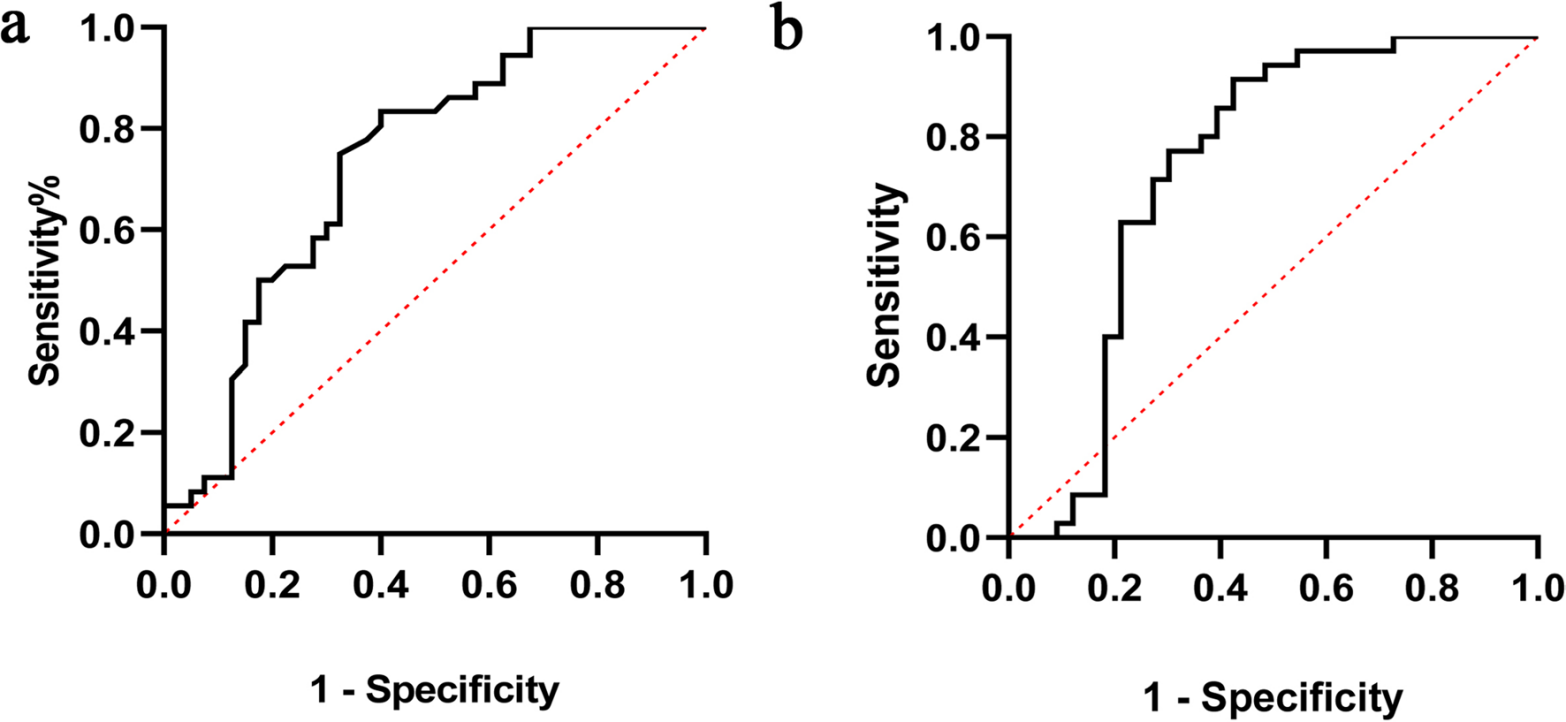
ROC curve analysis for determining statistically significant differences between study groups. (**a**) ROC curve of Sporadic patients and healthy controls analyzed for relative expression level of *VDR* (AUC: 0.73, p value: 0.0005). (**b**) ROC curve of Sporadic patients and HFR group analyzed for relative expression level of *CRB3* (AUC: 0.73, p value: 0.0008).

Diagnostic test evaluation was performed for genes whose expression had changed significantly in the defined groups, by ROC curve and AUC calculation. According to the expression level of *VDR* in the sporadic versus control group, AUC was 0.73 (95% CI 0.61–0.84; p-value: 0.0005) (Fig. 2a). In the diagnostic performance evaluation of the *CRB3* gene expression in the sporadic versus HFR group, AUC was 0.73 (95% CI 0.60–0.86; p-value: 0.0008) (Fig. 2b). For other defined groups, AUC did not change significantly; the ROC curves are provided in Supplementary Fig. S2.

### Assessment of the relative gene expression levels of *VDR*, *TAZ*, *YAP1*, and *CRB3* among patients and the control group

**Figure 3:**
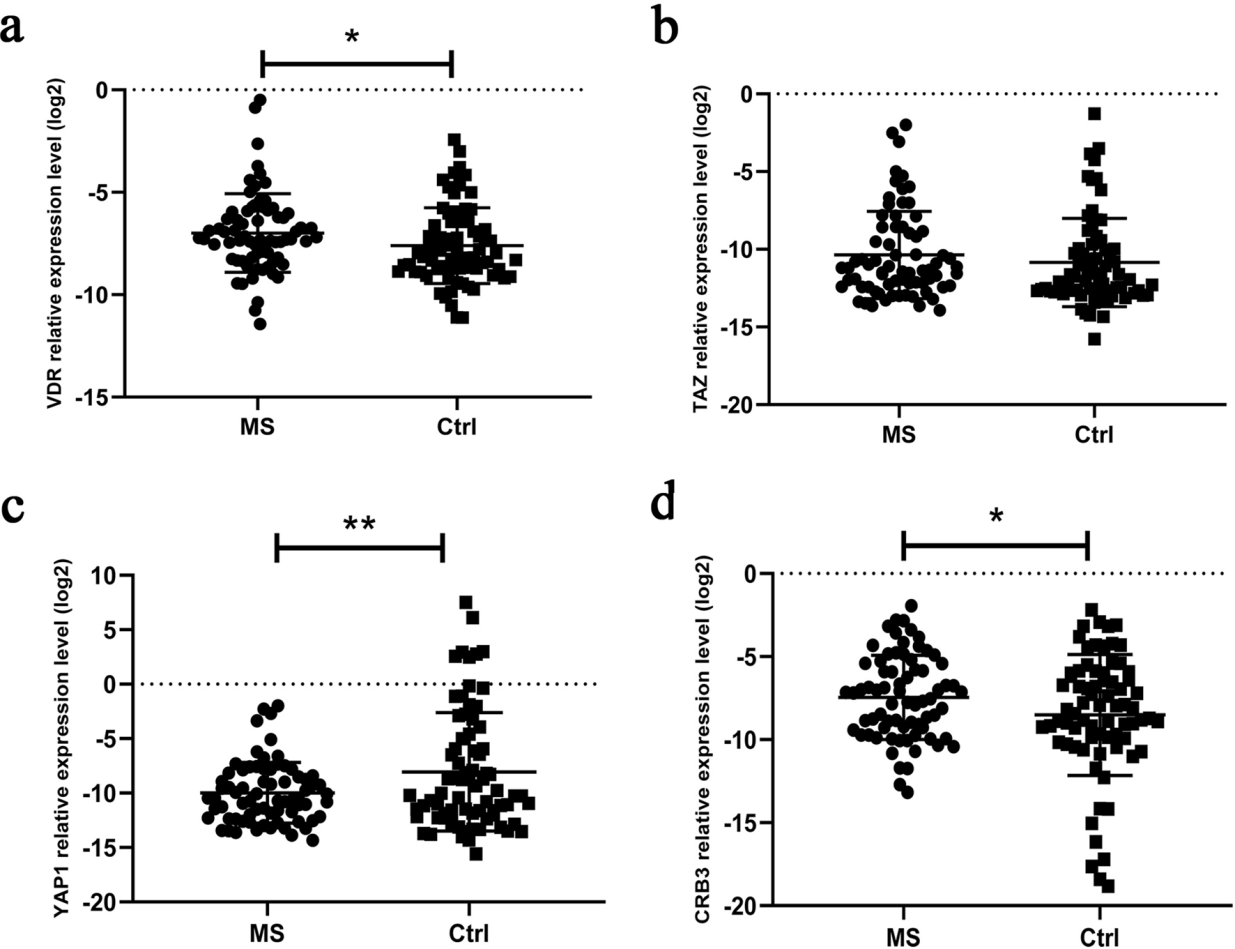
The relative expression level of (**a**) *VDR*, (**b**) *TAZ*, (**c**) *YAP1*, and (**d**) *CRB3* in all MS patients (n: 72) compared to healthy controls (n: 74). *MS* Multiple Sclerosis, *Ctrl* Control group. Whiskers represent the standard error of the mean. (*p < 0.05, **p < 0.01, ***p < 0.001).

In this part of the study, we grouped all MS patients (sporadic and familial groups) as clinically definite MS (n = 72), as a single group; also, all healthy individuals were taken as a definite control group (n = 74), the analyses were conducted accordingly. The relative gene expression level of *VDR* was higher among MS patients, as compared to the control group (p-value: 0.04, Fig. 3a). A similar difference in the *crb3* expression level existed among MS and control groups (p-value: 0.04, Fig. 3d), though it was not statistically significant in the *TAZ* gene expression (p-value: 0.30, Fig. 3b). Our results, therefore, showed that there was a significant decrease in the *YAP1* expression, as compared with the control group (p-value: 0.008, Fig. 3c).

Diagnostic test evaluation was also performed for genes whose expression had changed significantly in MS versus control group, by ROC curve and AUC calculation; however, there was no statistical significance. The ROC curves are provided in Supplementary Fig. S2.

### Association of *VDR*, *TAZ*, *YAP1, *and *CRB3* gene expressions with clinical parameters in the MS patients

When we explored the association of *VDR*, *TAZ*, *YAP1*, and *CRB3* relative expressions with EDSS and onset age of all patients as a single group, the results did not show any correlations. Later, we performed the correlation analyses in Sporadic and familial groups, separately. There were no significant correlations between *TAZ*, *YAP1*, and *CRB3* gene expressions and clinical parameters. However, a significant correlation was found between the *VDR* expression level and EDSS in the familial patients (r: 0.380, p-value: 0.032) (Table 2).

**Table 2:** Correlation coefficients (and p-values) of relative gene expressions with EDSS and Onset age in patient groups. Bold values denote statistical significance at the p < 0.05 level.

Variable	Covariate	Sporadic patients	Familial patients	All patients
Current EDSS	VDR relative expression (log2)	− 0.288 (0.094)	0.380 (**0.032**)	0.121 (0.328)
	TAZ relative expression (log2)	0.027 (0.877)	0.205 (0.261)	0.131 (0.289)
	YAP1 relative expression (log2)	− 0.099 (0.570)	− 0.116 (0.528)	− 0.072 (0.563)
	CRB3 relative expression (log2)	− 0.075 (0.669)	− 0.055 (0.765)	0.043 (0.728)
Onset age	VDR relative expression (log2)	− 0.001 (0.996)	0.251 (0.182)	0.159 (0.210)
	TAZ relative expression (log2)	0.030 (0.866)	0.139 (0.463)	0.087 (0.493)
	YAP1 relative expression (log2)	0.290 (0.096)	0.147 (0.438)	0.217 (0.084)
	CRB3 relative expression (log2)	0.318 (0.067)	0.165(0.385)	0.220 (0.081)

**Figure 4:**
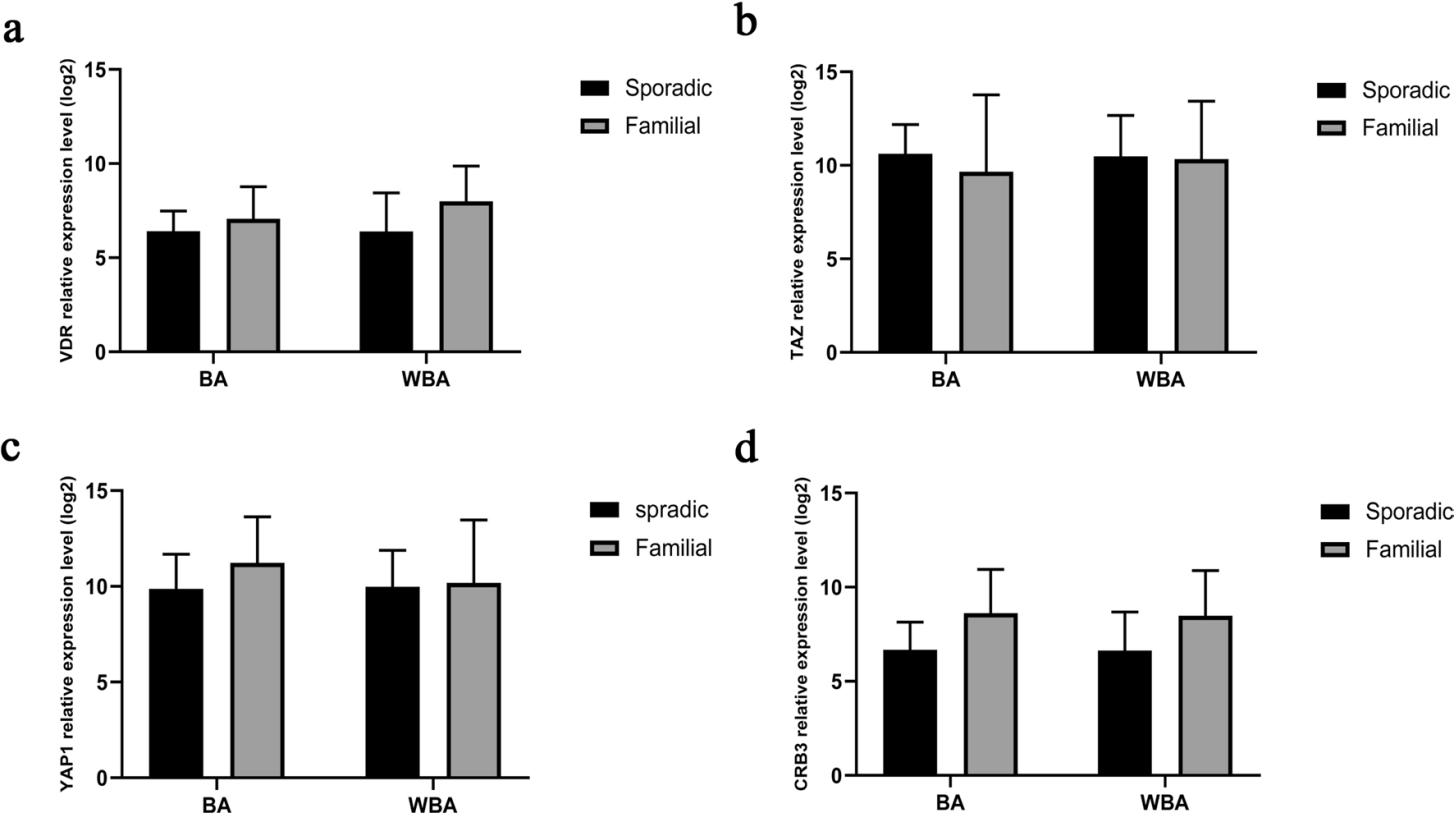
Comparison of gene expression of (**a**) *VDR*, (**b**) *TAZ*, (**c**) *YAP1*, and (**d)** *CRB3*, in the Familial and Sporadic patients with and without brain atrophy. The columns represent the mean ± SD. Data were analyzed by two‐way ANOVA followed by LSD post hoc test. *BA* brain atrophy, *WBA* without brain atrophy.

Additionally, the effect of brain atrophy on the relative gene expressions was evaluated in the sporadic and familial groups by two-way ANOVA, followed by the LSD post hoc test. None of the gene expressions in the Sporadic and familial groups was affected by brain atrophy (Fig. 4).

## Discussion

MS is a chronic inflammatory disease of the central nervous system. The most common effects include myelin destruction, varying degrees of inflammation, complement activation, oligodendrocyte death, axonal injury, and plaque formation in the brain^30,31^. The repair of myelin damage to prevent further axonal loss can prevent the disease before progressing to the acute phase; therefore, many studies are currently focused on overcoming the signaling barriers to remyelination by oligodendrocytes. Also, investigation of the molecular mechanisms involved in glial cell myelination may play an important role in understanding the factors involved in this myelination and their potential use as biomarkers in the diagnosis and even treatment of MS^32^. Due to the evidence indicating the association of *VDR*, *TAZ*, *YAP1*, and *CRB3* with demyelination process, we analyzed their gene expression in different groups of MS and healthy individuals to evaluate whether these molecules could contribute as biomarkers to familial and sporadic MS or become efficient, even in personalizing the treatment of the patients.

Previous studies have shown that in patients with MS, *VDR* gene expression is increased following vitamin D depletion to use the lowest levels of vitamin D. Under these conditions, oligodendrocyte progenitor cell differentiation is reduced. Therefore, damage to the myelin sheath of the axons will not be restored and the symptoms of the disease will intensify rapidly^33,34^. The results of comparing the gene expression in four groups of familial and sporadic MS patients and healthy individuals with and without the family history of the disease showed that the *VDR* gene was significantly increased in the sporadic patients, as opposed to the familial group, compared with both HFR and control groups. Consequently, it could be said that this increase in the *VDR* gene expression might be significantly correlated with sporadic MS cases. Some studies have suggested that the presence of various single nucleotide polymorphisms (SNPs) in the *VDR* gene affects its expression^35^. Therefore, it can be hypothesized that the reason for the difference in expression between familial and sporadic groups is the presence of different SNPs in one of these groups that have affected the *VDR* expression. The results also showed that the *VDR* gene expression in all patients was significantly increased, as compared to the healthy individuals, indicating a failure to repair the damaged myelin (remyelination) and serving as a reason for intensifying symptoms. Previously, *VDR* gene expression in different populations has been evaluated in patients versus healthy controls. In the Netherlands, for example, in 2013, Smolders et al. compared the expression of *VDR* among 39 MS patients and 20 healthy individuals. The results of their studies showed a significant increase (p < 0.01) in the expression of *VDR* in the brain lesions of the patients, as compared to the control group^36^. Comparison of the *VDR* gene expression in Spain, in 2017, also showed a 6.5-fold increase in the MS patients^37^. In a similar study, a significant decrease in the *VDR* gene expression after 2 months of treatment with vitamin D supplements was reported in MS patients^38^. In our study, although patients received vitamin D supplements and had normal serum levels, they still had higher levels of gene expression than controls. This can be related to the supplement dosage and treatment duration of the patients. Increased levels of the *VDR* gene may also be due to SNPs or miRNAs which may influence its expression. A synergistic effect between vitamin D and IFN-Beta is reported and the combination of vitamin D plus IFN-β reduced inflammatory cytokines and has potential benefit in ameliorating MS^39,40^. Thus, it is hypothesized that treatment with IFN-Beta, like vitamin D supplementation therapy, reduces the expression of the *VDR* over time. It seems that up-regulation of *VDR* in MS patients might have been even more prominent if we had excluded the effect of IFN-Beta on *VDR* expression.

Sadeghi et al. have also investigated the expression level of *VDR* in RR-MS patients in comparison with normal individuals in the Iranian population. Similarly, the patients demonstrated a significantly higher expression level of *VDR* than the control group (p = 0.04)^41^. *VDR* expression has also been studied in other neurological diseases. Mazdeh et al. evaluated the expression of *VDR* in epileptic patients. They found significant downregulation of *VDR* expression in patients and suggested that it has a pathogenic role in epilepsy through attenuation of the physiologic functions of vitamin D in neuroprotection or regulation of the immune system^42^. Besides, Balta et al. demonstrated a significantly higher *VDR* expression in autistic disorder patients compared to control subjects, that is in accordance with an increase in the innate immune response^43^.

There is, however, a lack of genetic evaluation in familial versus sporadic MS patients. We only found one research that investigated the relationship between familial MS patients present in a Turkish population and *VDR* genotypes Taq I, Apa I, and Fok I polymorphisms. Their findings suggested an association between *VDR* Taq I polymorphism and familial MS^44^. In addition, we found a significant correlation between the expression level of *VDR* and EDSS in familial patients. While this shows the potential impact of *VDR* overexpression in the clinical course of MS, due to the small number of progressive MS patients in our study population, it is difficult to introduce *VDR* as a biomarker that can reliably predict the MS disability progression. Therefore, this correlation should be confirmed in future studies with larger familial progressive MS patients.

The evaluation of the effect of vitamin D on Intrahepatic Cholangiocarcinoma (ICC) in 2016 showed that one of the nuclear effectors of the Hippo pathway, *TAZ*, was highly expressed in the ICC tissues. In this study, it was shown that the *TAZ* gene increased *VDR* and decreased p53 expression by decreasing the tumor cell susceptibility to vitamin D. Vitamin D could effectively down-regulate the expression of *VDR* and *TAZ,* and up-regulate the p53 expression, which might result in inhibiting cell proliferation of ICC and worsening or spreading the disease^25^. Observing the effect of *TAZ* on *VDR* as a positive regulator prompted us, for the first time, to investigate the expression of it in different groups of MS, as compared to healthy controls. Due to the up-regulation of *VDR* in MS patients, *TAZ* expression was also expected to increase. The expected high expression was seen in the present study, although it was not statistically significant. The expression of this gene was also significantly increased in the familial MS and HFR, as compared to the control group, thereby indicating a possible association of the *TAZ* expression with the familial MS. It seems that with increasing the sample size, increasing gene expression in familial compared to sporadic patients will be significant and will strengthen our hypothesis. Interestingly, a similar gene expression level among familial and HFR groups was seen, but gene expression in both groups had significantly increased compared to the control group. It seems that differential expression of this gene can be considered as a predisposing factor in HFRs. A majority of genetically predisposed individuals may remain asymptomatic for the rest of their lives or develop the disease due to various environmental factors. It seems, therefore, that the existence of a specific variant in all individuals of these families could lead to an increase in gene expression, in comparison to the healthy individuals without a family background. As mentioned before, different miRNAs also have the potential to affect the expression of a gene. Therefore, it is also possible that the *TAZ* expression in familial individuals has been affected in this way. Contradictory findings, however, indicate the suppression of *YAP1/TAZ* expression by *CRB3* in the Hippo pathway in myelinating glial cells, which is followed by demyelination in the nervous system^45,46^. Therefore, a decrease in *YAP1/TAZ* and an increase in *CRB3* expression in the MS patients were expected, although no studies had been performed to investigate the expression of the core components of the Hippo pathway in MS. Of course, there are studies available worldwide examining Hippo pathway effector genes in other neurological diseases. Mueller et al. investigated the possible role of this pathway in Huntington’s disease (HD) pathogenesis. Their results demonstrate a significant decrease in *YAP1* in neuronal stem cells derived from HD patients that could significantly contribute to transcriptional dysregulation in HD^47^.

Besides, Morimoto et al. demonstrated a decrease in the levels of YAPdeltaC, a neuronal isoform of YAP, in the spinal cords of Amyotrophic lateral sclerosis (ALS)-transgenic mice. They suggested that this could be correlated with disease progression in ALS animal models^48^.

In fact, the process of myelin sheath elongation by oligodendrocytes requires the *YAP1*, which is phosphorylated and inactivated by the *CRB3* when the Hippo pathway is on, thereby inhibiting the myelination or elongation of nerve cells. It is interesting that although inhibition of the Hippo pathway leads to tumor cell proliferation, its activation may play a role in neurodegeneration. In the present study, *YAP1* expression was significantly decreased in the MS patients as compared to controls, and *CRB3* expression was significantly increased. Thus, as expected, this reduced expression of the *YAP1* leads to incomplete elongation of the myelin sheath components and the pathology of MS disease. In this case, the presence of a drug or combination that eliminates the inhibitory effect of *CRB3* on *YAP1* activity or in other words, blocks the Hippo pathway may help form a long myelin sheath during remyelination and reduce the effects of the disease. A significant increase in the *YAP1* expression in the HFR group, as compared to the familial MS patients, could help to improve prognosis in the younger individuals. This hypothesis can only be accepted if HFRs are no longer supposed to develop the disease so that this increased gene expression can be introduced as a prognostic biomarker. Since we preferred older healthy first-degree relatives, the possibility of using this gene as a prognostic biomarker could be strengthened. However, further studies are needed to prove this hypothesis.

A similar gene expression profile for the *CRB3* was observed in the familial and HFR groups. Since gene expression in all patients was significantly higher than in healthy controls, the increased expression of *CRB3* in HFRs compared with controls would also be expected, so that changes in the expression of this gene could be introduced as a predisposing factor for MS. By contrast, the results showed a significantly decreased gene expression in the HFR group compared to controls, which refutes our hypothesis of identifying this gene as a predisposing factor.

This study was the first research to compare the expression profiles of Hippo pathway effector genes in MS with the genetic classification of disease into two different groups, familial and sporadic, and by involving healthy first-degree relatives of familial MS cases in the study. However, a limitation of this research was the small sample size of the participants. Additional studies in a larger cohort are needed to clarify if *YAP1, TAZ, CRB3*, and also *VDR* can be used as biomarkers for MS prognosis. Targeting other key genes of the Hippo signaling pathway such as *MST, LATS, TEAD, SAV1*, etc. in future studies will help confirm the involvement of this pathway in the pathogenesis of MS. Also, it is suggested that the study of the effect of Interferon-Beta on the expression level of Hippo pathway effector genes be the subject of future researches to help interpret the results of such studies.

## Conclusions

Overall, this research investigated the possible role of *YAP1, TAZ, CRB3*, and *VDR* as prognostic markers in MS patients. Many genetic tests nowadays use prognostic biomarkers to inform family members at risk and predict diseases before they are clinically expressed. The present study is an important step towards this direction. Additionally, the use of prognostic and predictive biomarkers has a great potential value for drug development, therapeutic decision-making for patients, and the decrease of medical costs. Confirmation of different gene expression patterns in the familial and sporadic MS patients in the future may have obvious implications for personalized pharmacological treatment and disease management.

## Supplementary Information

Supplementary Information.

## Data Availability

Data sharing not applicable to this article as no datasets were generated or analyzed during the current study.
