# Supplementary material for: Gene expression profiles of YAP1, TAZ, CRB3, and VDR in familial and sporadic multiple sclerosis among an Iranian population
Source: Sci Rep. 2021 Apr 8;11:7713. doi: 10.1038/s41598-021-87131-z (PMC8032816; doi:10.1038/s41598-021-87131-z)
Supplement: Supplementary file 1 — Supplementary Information. [file 41598_2021_87131_MOESM1_ESM.pdf]

Gene Expression Profiles of YAP1, TAZ, CRB3, and VDR in Familial and Sporadic Multiple Sclerosis Among an Iranian Population

Sheyda Khalilian[1], Zohreh Hojati[1*], Fariba Dehghanian[1], Vahid Shaygannejad[3], Seyedeh Zahra Hosseini Imani[1], Majid Kheirollahi[2], Mehdi Khorrami[2], Omid Mirmosayyeb[3]

1. Division of Genetics, Department of Cell and Molecular Biology and Microbiology, Faculty of Biological Sciences and Technologies, University of Isfahan, Isfahan, Iran.
2. Research Institute for Primordial Prevention of Non-Communicable Disease and Department of Genetics and Molecular Biology, School of Medicine, Isfahan University of Medical Sciences, Isfahan, Iran.
3. Isfahan Neuroscience Research Center, Isfahan University of Medical Sciences, Isfahan, Iran.

* Correspondence: Dr. Zohreh Hojati

Postal Code: 81746-73441,
Email: z.hojati@sci.ui.ac.ir

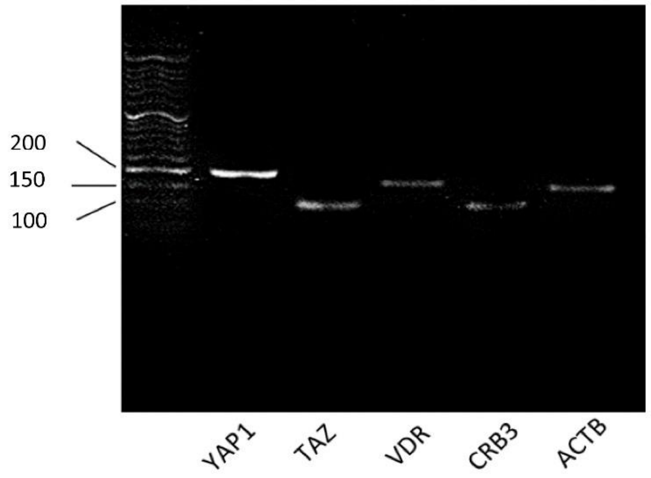

Supplementary Fig. 1 DNA gel electrophoresis for validation of gene amplification in RT-PCR. a patient sample run on a 2% agarose gel stained with Ethidium Bromide. The first line accommodates a 50 base bp ladder. Amplicon size of *YAP1* =196bp, *TAZ* =98bp, *VDR* =160 bp, *CRB3* =107bp, *ACTB* =151bp

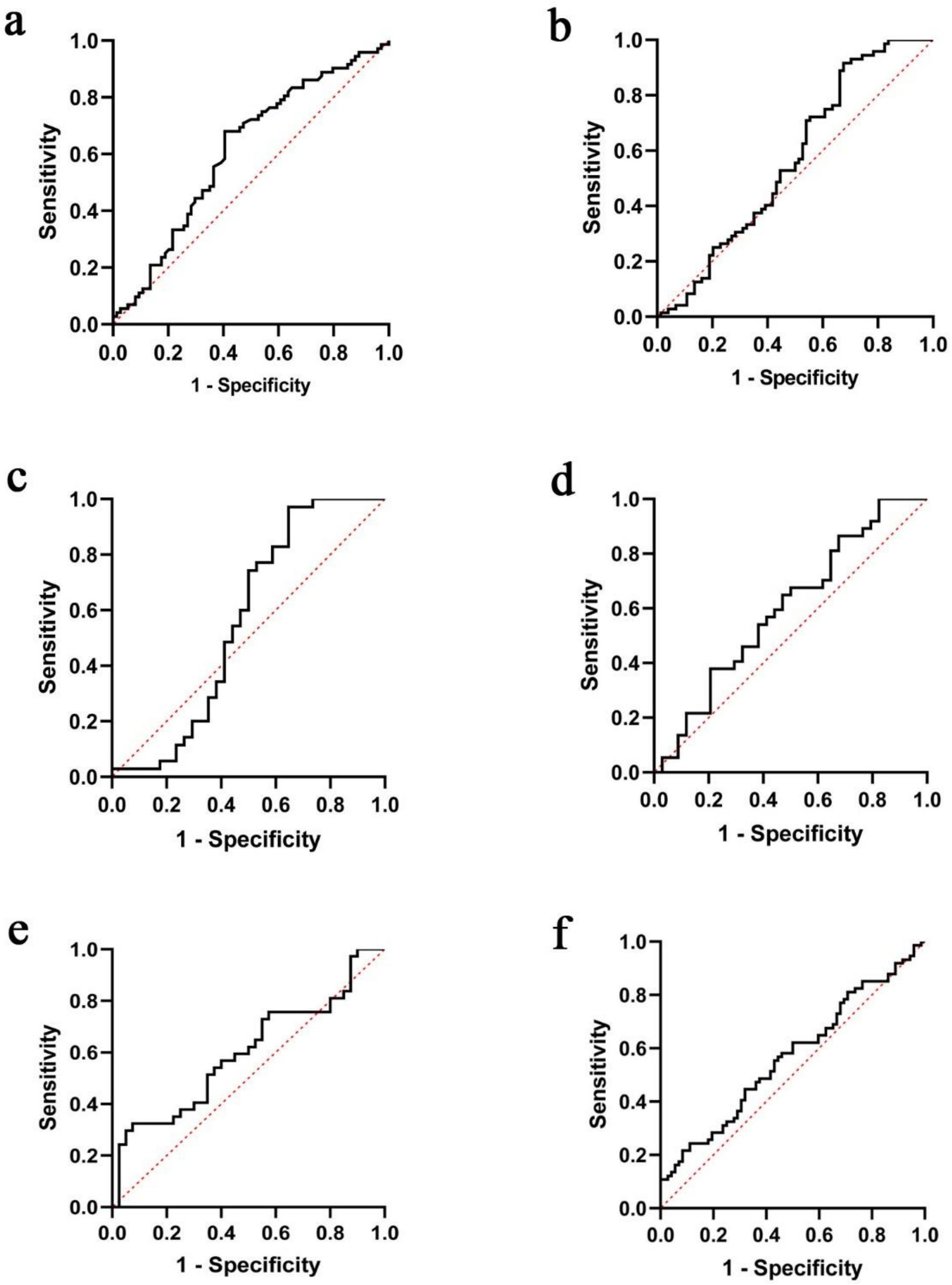

Supplementary Fig. 2 ROC curves for *VDR*, *TAZ*, *YAP1* and *CRB3*. **(a)** ROC curve of all patients and healthy controls analyzed for relative expression level of *VDR* (AUC: 0.61, p value: 0.02). **(b)** ROC curve of all patients and healthy controls analyzed for relative expression level of *YAP1* (AUC: 0.56, p value: 0.16). **(c)** ROC curve of Sporadic patients and HFR groups analyzed for relative expression level of *YAP1* (AUC: 0.56, p value: 0.38). (**d)** ROC curve of Familial patients and HFR groups analyzed for relative expression level of *YAP1* (AUC: 0.59, p value: 0.15). **(e)** ROC curve of Familial patients and healthy controls analyzed for relative expression level of *TAZ* (AUC: 0.60, p value: 0.12). (**f)** ROC curve of all patients and healthy controls analyzed for relative expression level of *CRB3* (AUC: 0.56, p value: 0.15).

Supplementary Table 1. List of primer couples generated for q-PCR

Gene	Forward primer	Reverse primer	Product length
**ACTB**	5'TTCGAGCAAGAGATGGCCA3'	5'CACAGGACTCCATGCCCAG3'	151 bp
**VDR**	5'CCCAACTCCAGACACACTCCCA3'	5'GGGACAGCTCTAGGGTCACAGA3'	160 bp
**TAZ**	5'CCCTCATCACCGTGTCCAA3'	5'ACGCATCAACTTCAGGTTCCA3'	98 bp
**YAP1**	5'ACAATGACGACCAATAGCTCAGAT3'	5'AACGGTTCTGCTGTGAGGG3'	196 bp
**CRB3**	5'CTGCAAATGAGAATAGCACTGT3'	5'CAAGAGGGAGAAGACCACGA3'	107 bp
